# Evaluation of the aerodynamic performance of the counter rotating turbo fan COBRA by means of experimental and numerical data

**DOI:** 10.1007/s13272-021-00565-z

**Published:** 2021-12-24

**Authors:** Thibault Ly, Kazim Koc, Lionel Meillard, Rainer Schnell

**Affiliations:** German Aerospace Center (DLR), Linder Höhe, Cologne, Germany

**Keywords:** Aeronautics, Propulsion, Aerodynamics, Counter rotating turbo fan, Validation, Measurement uncertainties, Torque, EU–Russian collaboration

## Abstract

In the present study, steady numerical simulations performed on the counter rotating turbo fan (CRTF) COBRA are compared with experimental data carried at the CIAM C-3A test-bench in Moscow. For this purpose, a systematic analysis of the measurement uncertainties was performed for the global aerodynamic performances of the CRTF, namely, the massflow, the total pressure ratio, the isentropic efficiency, as well as the torque ratio applied on both fan rows. Several numerical models are investigated to highlight their effects on the aforementioned predicted quantities. Differences in modeling consist in grid resolutions and the use of two turbulence models popular in the turbomachinery community. To match as much as possible the experiment running conditions, the performance map of the CRTF is simulated using the exact measured speed ratio and massflow. The comparisons show good estimations of the numerical simulation over the entire performance map. The main differences between the turbulence models occur at part-speed close to stall conditions. More surprisingly at aerodynamic design point, the importance of the turbulence modeling on the predicted torque ratio has been pointed out.

## Introduction

**Fig. 1:**
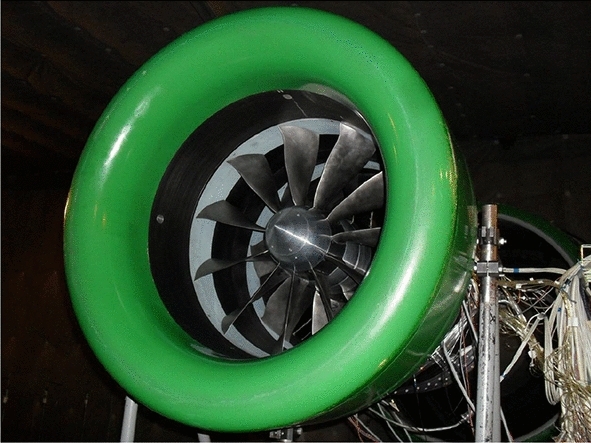
CRTF COBRA mounted at the CIAM test-rig in Moscow (picture taken by CIAM)

**Fig. 2:**
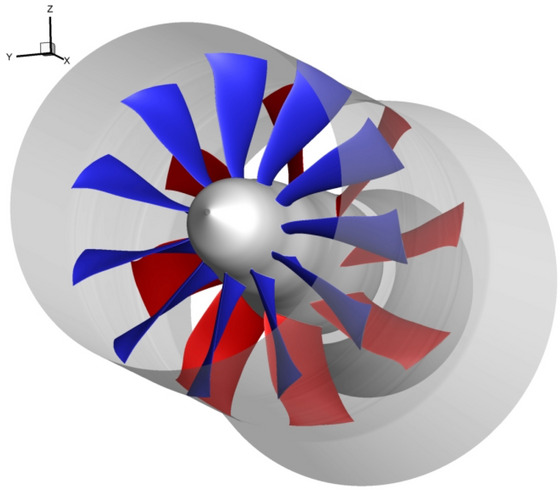
Numerical view of the CRTF COBRA with blades of the front fan (blue) and for the rear fan (red)

The annual number of airplane passengers was expected to practically double in the coming 20 years. Indeed, in 2017, the Air Transport Action Group (ATAG) estimated that number at 4.1 billion [1]. According to the International Air Transport Association (IATA), it was supposed to become 7 billion by 2037 [2]. However, because of the COVID-19 pandemic, these forecasts needed to be reviewed. Shortly after the pandemic breakthrough and according to [3], three possible scenarios impacting the volume of the passengers’ air traffic have been examined. A rebound may have to recover the volume of the pre-pandemic period by winter 2020, a delayed cure scenario should have to recover 90% of that volume by summer 2021, and a recession might enable to only recover 80% of that volume by summer 2022. One year on from the pandemic began, a more pessimistic scenario, that seems to be emerging, forecasts a number of passengers per year is not expected to return to its pre-COVID-19 level before 2024 [4]. Still, the demand will increase sooner or later; therefore, more efficient and sustainable aircraft need to be designed in regards to the environmental crisis that the world is facing. The Advisory Council for Aviation Research and innovation in Europe (ACARE) was already addressing this problematic. In 2001, it set emissions targets for year 2020 in the report: “European aeronautics: a vision for 2020” [5]. Namely, a 50% cut in CO_2_ emissions per passenger-kilometer and an 80% cut in NO_x_ emissions, in comparison to the capabilities of typical new aircraft from year 2000, were expected. In 2011, these goals were updated in the report: “Flightpath 2050 Europe’s Vision for Aviation” [6]. By 2050, it is expected that the future airplanes will reduce the previous cited emissions to, respectively, 75% and 90%, also in comparison to year-2000 aircrafts. To cope with these objectives, the counter rotating turbo fan appears as a solution that has potential in comparison to a single rotating fan, where the stator is replaced by a fan which is rotating in the opposite direction of the front fan [7–10]. As a consequence, the flow at the exit of the compression is less swirled and there are fewer losses in the rest of the engine. It could result in a 1.8% efficiency improvement than a single fan architecture, for the same level of compression [11].

**Fig. 3:**
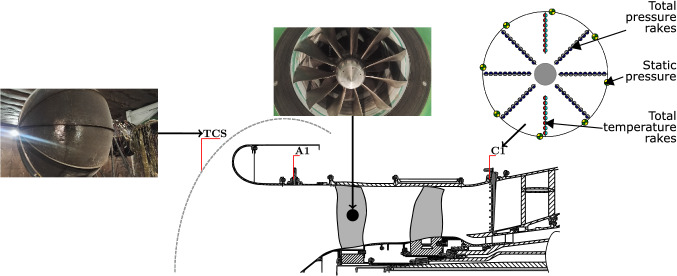
Test-rig cross-section displayed with the TCS (left-hand side) and the COBRA fans (top) mounted during the test campaign at the CIAM including circumferential rakes positions and radial arrangement of measurement probes at section C1 (right-hand side)

The project counter rotating fan system for high bypass ratio aircraft engine (COBRA) aims at integrating that technology into a ultra-high bypass ratio (UHBR) ducted engine for a short/medium-range aircraft. The philosophy behind the project is to cumulate potential benefits in efficiency by combining the CRTF and the bypass technologies, and at the same time to reduce the perceived noise thanks to the bypass flow [12, 13]. With the support of the European Commission Seventh Framework Programme, the project started in 2013 and ended in 2018. A parallel goal was to reinforce the EU-Russian relations in the aeronautic industry. Therefore, in the COBRA project: SAFRAN was responsible for the specification and baseline design delivery, the DLR for the conception and optimization of the fans geometry (Fig. 1) and also of the postprocessing of the data from the experimental campaign, COMOTI for the manufacturing, the CIAM for the experimental campaign (Fig. 2), and the ONERA for the lead and support of the project. The aerodynamic performance of the COBRA fans was set in regards to its predecessor the CRTF VITAL project [14], which was also conducted by an EU–Russian collaboration. Namely, the target is to maintain the isentropic efficiency performance but with a higher bypass ratio (> 15). Thus, both the fan pressure ratio and the noise level could substantially be lowered. In the present paper, since the study focuses on the aerodynamics, the acoustic objectives are not tackled nor mentioned. To decouple the rotating speeds of both the front and rear fans, a planetary differential gearbox was used [15]. The gearbox implied that the torque ratio has a constant value. This restriction was fulfilled during both the design and optimization processes, and the experimental tests. That restriction is the reason why the operating limits of the COBRA fans were tested with iso-torque lines. All the flight phases were tested by guarantying that the ratio of the torque of the rear fan over the torque of the front fan was constant. To do so, the rotating velocity of the front fan was fixed, according to each flight phase, while the massflow penetrating into the CRTF was modified by the suction system at the outlet of it. To keep the torque ratio fixed, the rotating velocity of the rear fan was adapted. That method enabled to test the CRTF COBRA at several operating points, which triggered the creation of the so-called iso-torque lines. In the present paper, the rotating speed of the front fan is taken as a reference to designate to which operating condition the CRTF is running at.

After the experimental campaign, a lot of experimental data were available. Indeed, the CRTF COBRA was tested for six different running conditions. However, there were no numerical studies that investigate the operability limits, as it was performed during the experimental campaign. Therefore, the present study enables to investigate the numerical capabilities of the DLR tools, to supplement the CRTF validation programme, and enhance the knowledge on CRTF aerodynamics. The first section focuses on the measurement methodology used to assess the aerodynamic output quantities. Thanks to that study, the links between the measurements and the meaningful experimental output quantities are enlightened. In addition, an uncertainties study is carried out on the experimental quantities with a statistical approach, to enhance the validation purpose [16]. The second section presents the numerical method used to simulate the performance of the fans in the same corrected conditions as in the experimental campaign. Therefore, the grid resolutions used to perform medium and high fidelity simulations, the boundary conditions, the equations, and assumptions taken into consideration by the flow solver and the postprocessing are presented. The final section is an analysis and a comparison between the experimental data and numerical results regarding the COBRA aerodynamic performances, with the help of average quantities and radial distributions.

## Experimental campaign

**Table 1:** Relative radial positions of total pressure probes at section C1

$R_{1}/R_{10}$	$R_{2}/R_{10}$	$R_{3}/R_{10}$	$R_{4}/R_{10}$	$R_{5}/R_{10}$
0.47	0.51	0.55	0.63	0.70

$R_{6}/R_{10}$	$R_{7}/R_{10}$	$R_{8}/R_{10}$	$R_{9}/R_{10}$	$R_{10}/R_{10}$
0.78	0.85	0.92	0.97	1.00

**Table 2:** Relative radial positions of total temperature probes at section C1

$R_{1}/R_{8}$	$R_{2}/R_{8}$	$R_{3}/R_{8}$	$R_{4}/R_{8}$
0.48	0.56	0.63	0.71

$R_{5}/R_{8}$	$R_{6}/R_{8}$	$R_{7}/R_{8}$	$R_{8}/R_{8}$
0.78	0.85	0.92	1.00

The experimental campaign was held at the CIAM test-rig C-3A. The 1150 ${\mathrm{m}}^{3}$-room is an anechoic chamber that enables to obtain aerodynamic and acoustic measurements. Figure 3 is a cross-section illustration of the test-bench located at the CIAM. On the left-hand side, a turbulence control screen (TCS), placed at the intake of the test-rig, allows reducing the turbulence effects and enables a smooth air flow to penetrate the test-rig. There are two axial positions where temperature and pressure are measured. First, at section A1, there are 16 static pressure probes on the casing. The second measurement position is located at section C1, in the Bypass passage. There are on each the casing and the upper surface of the splitter eight static pressure probes, on the vertical symmetry plane two rakes of total temperature probes and in the rest of Bypass passage six rakes of total pressure probes. On the right-hand side of Fig. 3, the rakes and probes positions at section C1 are depicted. This placement enables to collect data in the Bypass passage. Each total pressure rake has ten probes and each total temperature rake has eight thermo-element probes (thermocouple Type K). The relative radial positions of the probes on both the total pressure and total temperature rakes are given, respectively, in Tables 1 and  2. Achieved by the CIAM, a data processing consisted in time averaging of 30 records for a single operating point and, whoever is needed, in spacial averaging. The experimental results are presented latter in Sect. 4.

### Measurement methodology and calculation of the output quantities

**Fig. 4:**
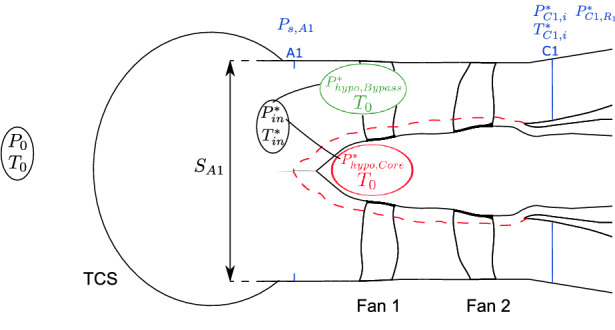
Aerodynamic measurements locations at the CIAM C3-A test-bench

The experimental set-up described in the previous section enables to measure pressure and temperature input quantities. The objective of this section is to highlight the equations used to assess relevant aerodynamic output quantities and to know how they derive from measurement inputs. Then, thanks to this analysis, uncertainty study could be carried out on those aerodynamic output quantities. Therefore, the present study focuses on seven output quantities: the corrected massflow ${\dot{m}}_{\mathrm{cor}}$, the total pressure ratio $\Pi _i^*$, the aerodynamic isentropic efficiency $\eta _{\mathrm{is,aero},i}$, the average total pressure ratio $\Pi ^*$, the average aerodynamic isentropic efficiency $\eta _{\mathrm{is,aero}}$, the mechanical isentropic efficiency $\eta _{\mathrm{is,mech}}$, and the torque ratio $\tau$. Figure 4 is a scheme of the test-rig configuration with the locations of the experimental input quantities. These quantities are to be detailed when they are used in the expression of the five output quantities.

*Corrected massflow* ${\dot{m}}_{\mathrm{cor}}$ The corrected massflow ${\dot{m}}_{\mathrm{cor}}$ is assessed, for each operating point, thanks to the massflow *m*, the International Standard Atmosphere (ISA) at sea level conditions $(P_{\mathrm{ISA}}, T_{\mathrm{ISA}})$, and the measured conditions at the inlet $(P_{\mathrm{in}}^*,T_{\mathrm{in}}^* )$ [17, 18]. The relation between these quantities is defined as in Eq. (1)

1 $$\begin{aligned} {{\dot{m}}}_{\mathrm{cor}}={\dot{m}}\frac{P_{\mathrm{ISA}}}{P_{\mathrm{in}}^*}\sqrt{\frac{T_{\mathrm{in}}^*}{T_{\mathrm{ISA}}}}. \end{aligned}$$

There are at this point three unknowns, the massflow and the conditions of pressure and temperature at the inlet. The massflow sucked by the COBRA test-rig is assessed with the continuity Eq. (2). It is assumed that the air is an ideal gas and that the transformations that occur along the fans compression are isentropic

2 $$\begin{aligned} { {{\dot{m}}} =S_{\mathrm{A1}}M_aP_0\sqrt{\frac{\gamma }{r{\ T}_0}}\left( 1+\frac{\gamma -1}{2}M_a^2\right) ^\frac{1+\gamma }{2(1-\gamma )}K}. \end{aligned}$$

The surface of section A1 is denoted $S_{\mathrm{A1}}$. The total temperature $T_0$ and the total pressure $P_0$ are, respectively, the mean of total pressure and total temperature measurements taken at three different positions in the C-3A anechoic chamber (ambient condition). To calculate the actual massflow which deviate from the ideal massflow due to boundary layers or flow streamline curvature, the discharge coefficient, $K(P_{\mathrm{s,A1}},P_0)$, is estimated thanks to the static pressure measurements made at section A1. $P_{\mathrm{s,A1}}$ is the average value of the 16 static pressure measurements. The Mach number is expressed with the isentropic equation, and therefore, it depends on the pressure quantities, so $M_a(P_{\mathrm{s,A1}},P_0)$. The value of $T_{\mathrm{in}}^*$ is assumed to be the one of the average temperatures in the anechoic chamber $T_0$, so $T_{\mathrm{in}}^*=T_0$. To evaluate the total pressure at the fan inlet $P_{\mathrm{in}}^*$, the flow is conceptually divided into two parts: one part which goes through the Core and the other one which goes through the Bypass. This hypothetical division of the flow is written as in Eq. (3) and it is depicted in Fig. 4

3 $$\begin{aligned} {P_{\mathrm{in}}^*=\frac{(P_{\mathrm{Hypo,Core}}^*{{\dot{m}}}_{\mathrm{Core}}+P_{\mathrm{Hypo,Bypass}}^*{{\dot{m}}}_{\mathrm{Bypass}})}{{\dot{m}}}}. \end{aligned}$$

The Core massflow ${\dot{m}}_{\mathrm{Core}}$ is measured thanks to a Venturi duct system and the Bypass massflow is deduced as follows: ${{\dot{m}}}_{\mathrm{Bypass}}={\dot{m}}-{{\dot{m}}}_{\mathrm{Core}}$. The value of $P_{\mathrm{Hypo,Core}}^*$ is assumed to be the value of the average pressure in the anechoic chamber: so, $P_{\mathrm{Hypo,Core}}^*=P_0$. The value of $P_{\mathrm{Hypo,Bypass}}^*$ is estimated, thanks to the average static pressure at section A1 and losses coefficients (not detailed here) which take into account losses from the inlet of the test-rig up to the leading edge of Fan 1. It results in $P_{\mathrm{Hypo,Bypass}}^*(P_{\mathrm{s,A1}},\ P_0)$. Finally, the massflow, the pressure, and temperature conditions at the inlet are known. Therefore, the corrected massflow ${{\dot{m}}}_{\mathrm{cor}}$ is now assessable and the link to the measurement quantities is clear, so the functional relationship could be written down as in Eq. (15).

*Total pressure ratio* $\Pi _{i}^*$ The total pressure ratio is estimated thanks to the total pressure at the inlet $P_{\mathrm{in}}^*$ and the measurements of the total pressure $P_{\mathrm{C1},i}^*$. A circumferential average is performed over the six total pressure rakes and it ends up with ten values. One value of $P_{\mathrm{C1},i}^*$ for each radial position (as the index ‘*i*’ indicates). Finally for each operating point, there are ten values of the total pressure ratio $\Pi _i^*$, also one for each probe radial position from a total pressure rake in section C1, which are calculated thanks to Eq. (4). The functional relationship of the total pressure ratio is written in Eq. (16)

4 $$\begin{aligned} {\Pi _i^*=\frac{P_{\mathrm{C1},i}^*}{P_{\mathrm{in}}^*}}. \end{aligned}$$

*Aerodynamic isentropic efficiency* ${\eta }_{\mathrm{is},\mathrm{aero},{i}}$ The aerodynamic isentropic efficiency is estimated thanks to the parameters at the inlet $\left( P_{\mathrm{in}}^*,T_{\mathrm{in}}^*\right)$ and the ones measured by the rakes at section C1 $\left( P_{{\mathrm{C1}},i}^*,T_{{\mathrm{C1}},i}^*\right)$. To calculate $\eta _{\mathrm{is,aero},i}$, the quantities $P_{\mathrm{C1},i}^*$ and $T_{\mathrm{C1},i}^*$ must be known at the same radial position. However, the probes of both total pressure and total temperature rakes do not have the same radial positions (see Tables 1, 2). Therefore, first, a linear extrapolation is made on $T_{\mathrm{C1},i}^*$ to create two additional virtual values of total temperature. This process enables to extrapolate the total temperature values at the same radial positions as the total pressure measurements. Second, a circumferential average is also performed over those two temperature rakes and it also ends up with ten different values of $T_{\mathrm{C1},i}^*$ (as the index ‘*i*’ indicates). Finally, for each radial position of Table 1, there is a single value for both $P_{\mathrm{C1},i}^*$ and $T_{\mathrm{C1},i}^*$. For each operating point, this procedure enables to assess ten values of $\eta _{\mathrm{is,aero},i}$, with Eq. (5). The functional relationship of the aerodynamic isentropic efficiency, as written in Eq. (17), is nearly the same as the total pressure ratio one, except that the total temperature measurements at section C1 are added to it

5 $$\begin{aligned} {\eta _{\mathrm{is,aero},i} =\frac{{\Pi _i^*}^\frac{\gamma -1}{\gamma }-1}{\frac{T_{\mathrm{C1},i}^*}{T_{\mathrm{in}}^*}-1}}. \end{aligned}$$

The total pressure ratio $\Pi _i^*$ and the aerodynamic isentropic efficiency $\eta _{\mathrm{is,aero},i}$ are useful to analyze the performance of the CRTF over the height of the Bypass passage. Thus, experimental and numerical radial distributions may be compared. Whereas, the average total pressure ratio $\Pi ^*$ and the average aerodynamic isentropic efficiency $\eta _{\mathrm{is,aero}}$ enable to have a global idea of the CRTF performances. The method and the equations used to obtain average quantities, from their respective radial distribution, are detailed in the next subsections

6 $$\begin{aligned}&\eta _{\mathrm{is,mech}} \nonumber \\&\quad = \frac{c_{\mathrm{p}}T_{\mathrm{in}}^*\left( \left( {\frac{P_{\mathrm{C1,R1}}^*}{P_{\mathrm{in}}^*}}^\frac{\gamma -1}{\gamma }-1\right) \cdot {{\dot{m}}}_{\mathrm{Core}} +\left( {\Pi ^*}^\frac{\gamma -1}{\gamma }-1\right) \cdot {{\dot{m}}}_{\mathrm{Bypass}}\right) }{\frac{M_1\omega _1+M_2\omega _2}{i}\cdot (1-\varDelta )}. \end{aligned}$$

*Average total pressure ratio* ${\Pi }^*$ To estimate the average total pressure ratio, an entropy-based method was used. Thus, the average efficiency value is not affected by the total pressure losses. As written in Eq. (7), such a method implies the consideration of temperature measurements in the evaluation of the total pressure ratio

7 $$\begin{aligned} \Pi ^*=\exp {\left[ \frac{\sum \nolimits _{i=1}^{10}\left( \ln {\left( \frac{\Pi _i^*}{\left( \theta _i^*\right) ^\frac{\gamma }{\gamma -1}}\right) } {\delta {\dot{m}}}_i \right) }{\sum \nolimits _{i=1}^{10}{\delta {\dot{m}}}_i}\right] }\cdot \left( \theta _{\mathrm{av}}^*\right) ^\frac{\gamma }{\gamma -1}. \end{aligned}$$

With $\delta {{\dot{m}}}_i$ the infinitesimal massflow, $\theta _i^*=\frac{T_{\mathrm{C1},i}^*}{T_{\mathrm{in}}^*}$ the total temperature ratio, and $\theta _{\mathrm{av}}^*=\frac{T_{\mathrm{C1}}^*}{T_{\mathrm{in}}^*}$ the average total temperature ratio. A more classic method of massflow averaging has been performed for the total temperature. The massflow average enables to take into account the proportion of mass that is going through each probe domain of study. The averaging method of the total temperature is performed thanks to Eq. (8)

8 $$\begin{aligned} {T_{\mathrm{C1}}^*=\frac{\sum \nolimits _{i=1}^{10}{T_{C1,i}^*{\delta {\dot{m}}}_i }}{\sum \nolimits _{i=1}^{10}{\delta {\dot{m}}}_i}}. \end{aligned}$$

*Average aerodynamic isentropic efficiency* $\eta _{{\mathrm{is}},{\mathrm{aero}}}$ The estimations of the average total pressure ratio $\Pi ^*$ and the average of temperature $T_{\mathrm{C1}}^*$, in the previous subsection, enable to calculate the average global aerodynamic isentropic efficiency with Eq. (9)

9 $$\begin{aligned} {\eta _{\mathrm{is,aero}}\ =\frac{\left( \Pi ^*\right) ^\frac{\gamma -1}{\gamma }-1}{\frac{T_{\mathrm{C1}}^*}{T_{\mathrm{in}}^*}-1}}. \end{aligned}$$

In addition to the aerodynamic isentropic efficiency, the CIAM had also evaluated the isentropic efficiency based on torque measurements. Indeed, the CIAM performed a theoretical dependence of the isentropic efficiency accuracy in regards to both pressure and temperature accuracies. For a total pressure ratio of 1.1, if the total pressure measurements have an accuracy of 0.1%, then there is an accuracy of 0.1% on the isentropic efficiency value. And if the total temperature measurement has an accuracy of $0.5\,^{\circ }\hbox {C}$, then there is an accuracy of more than 5% on the isentropic efficiency value. For higher total pressure ratio, the accuracy of pressure measurement has roughly the same impact, but the influence of the accuracy of temperature measurement sharply decreases. Indeed, for a total pressure ratio of 1.6, if the total pressure measurement has still an accuracy of 0.1%, then there is an accuracy of 0.09% on the isentropic efficiency value. If the total temperature measurement has again an accuracy of $0.5\,^\circ \hbox {C}$, then there is an accuracy of 1% on the isentropic efficiency value. These observations justify why it was decided to evaluate the isentropic efficiency by mechanical means and not to rely only on aerodynamic measurements. The details on how torque measurements were used to determine the isentropic efficiency are given in the next subsection.

*Mechanical isentropic efficiency* $\eta _{\mathrm{is},\mathrm {mech}}$ The expression of the mechanical isentropic efficiency is written in Eq. (6). It relies on the torque measurements that enable to estimate the actual work consumed by the stage shaft and the idea that the flow is conceptually divided into a Core passage and a Bypass passage. Since there is no rake in the Core passage, it is not possible to measure the total pressure there. The assumption made is that the total pressure value at the minimum radial position of rake C1 ($R_1$, see Table 1) is the value of total pressure in the Core passage, written here $P_{C1,R1}^*$ and highlighted in Fig. 4. Here, $\omega _1=2\pi n_1$, $\omega _2=2\pi n_2$ and $M_1$ and $M_2$ are, respectively, the torque measurements at Fan 1 and Fan 2, *i* is the reduction coefficient of the shaft, and $\varDelta$ is the power losses in the shaft line. Therefore, thanks to the torque measurements, it is possible to estimate the isentropic efficiency without using the measurements of total temperature at the rakes of section C1. Therefore, in this study, the mechanical isentropic efficiency is the one plotted in global performance maps.

*Torque ratio* $\tau$ The torque ratio is defined, with the measurements of the torque at Fan 1 and 2, as in Eq. (10). The functional relationship of the torque ratio is written in Eq. (18)

10 $$\begin{aligned} {\tau =\frac{M_2}{M_1}}. \end{aligned}$$

### Experimental uncertainties’ study

The reliability of results of measurements to give the correct values of the physical quantities needs to be investigated. This doubt, which lies in any result of measurements, is named the uncertainty and gives an indication on the quality of the experimental results. For comparison purpose between experimental and CFD data, it is highly important to estimate the uncertainty of measurements. Indeed, the goal of the present study is to validate numerical results to experimental ones, so the experimental results are taken as references. To do so, one might know to what extend those references are well assessed.

#### Standard uncertainty principles

According to [19], one possible way to estimate the uncertainty is to use a probability distribution with the analysis of the standard deviation of the particular quantity. Therefore, in this approach, the uncertainty is now called a standard uncertainty, where the estimation of an output measurand *y* depends on determination of *N* input quantities $x_i$. The relation between the output measurand *y* and its *N* particular inputs $x_i$ is given by the functional relationship *f* in Eq. (11)

11 $$\begin{aligned} {y=f(x_1, x_2, \ldots , x_N)}. \end{aligned}$$

This relationship states that to evaluate the uncertainty of the measurand *y*, it is at first needed to evaluate the uncertainty of each input quantities $x_i$.

#### Type B standard uncertainty

The method used to estimate uncertainties of the COBRA project is the Type B, relying on a uniform distribution. In this method, the input quantity $x_i$ varies randomly between the bounds $a_-$ and $a_+$, and the probability that $x_i$ lies outside this interval is zero. The midpoint of the interval $\mu _i$, as defined in Eq. (12), is the expected value of $x_i$

12 $$\begin{aligned} {\mu _i=\frac{a_++a_-}{2}}. \end{aligned}$$

Thanks to those definitions, it is possible to estimate the Type B standard uncertainty of the uniform distribution as defined in Eq. (13)

13 $$\begin{aligned} {u\left( x_i\right) =\frac{{(a}_+-a_-)}{2\sqrt{3}}}. \end{aligned}$$

**Fig. 5:**
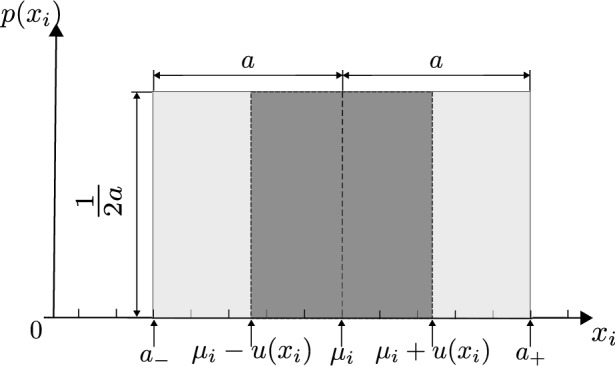
Graphical illustration of an uniform distribution

Figure 5 depicts the uniform distribution and the Type B standard uncertainty of $x_i$, with $a=\frac{a_+-a_-}{2}$. Finally, thanks to the uniform distribution, the uncertainties of each input quantity estimate could be calculated. These uncertainties are then combined to evaluate the expanded uncertainty of the output measurand.

#### Estimation of the COBRA project uncertainties

The corrected massflow, the total pressure ratio, the aerodynamic isentropic efficiency, and the torque ratio are the output measurands that were prioritized. The expressions of those output quantities are recalled in Sect. 2.1. Those expressions are in fact the functional relationships, previously named f, which link the input measurements to the output measurands. They are computed in the software GUM Workbench to calculate the associated expanded uncertainties [20].

*Temperature input quantity* For a thermo-element sensor of type K, the half-width interval of measurement is taken from [21]. In this document, the German accreditation administration stipulates that the use of a direct display thermometer with a temperature transmitter enables to measure the temperature with a ±0.2 K interval (within the temperature condition $-80\,^{\circ }\mathrm{C}<T<200\,^{\circ }$C). With the notations introduced previously, it is possible to calculate the upper border and the bottom border, namely $a_+ = T + 0.2$ K and $a_- = T - 0.2$ K. Then, the standard Type B uncertainty of the temperature quantity is calculable.

*Pressure input quantity* The CIAM stated that the measurement of the pressure is different in regards of the running conditions. At high mode, the measurement of the pressure has a $\pm 0.12\%$ interval, and at low mode, it has a $\pm 0.5\%$ interval. For the present study, it was decided to take the $\pm 0.5\%$ to cover all the possible uncertainties in pressure. Thus, the upper border and the bottom border are calculable; namely $a_+ = 1.005 P$ and $a_- = 0.995 P$. With the borders’ estimations, the Type B standard uncertainty for the pressure input quantity is now calculable.

*Diameter input quantity* The diameter is also an input quantity; it appears in the expression of the corrected massflow through the surface of section A1 (see Eq. 2). According to the structure and manufacturing departments knowledge and capability from the DLR, the design of such pipe comes with a 0.2 mm half-width of interval regarding the diameter.

*Core massflow input quantity* According to [22], a Venturi duct system with an inlet cone measures the massflow with an interval of $\pm 1\%$. This value is commonly used at the DLR while dealing with Venturi duct. Thanks to this information, both the borders of the core massflow measurement and the uncertainty on core massflow are calculable.

**Table 3:** Corrected massflow, total pressure ratio, aerodynamic isentropic efficiency, and torque ratio expanded uncertainty values (in %) at the probe $R_{5}$ for the 100% and 55% iso-torque lines

Speed (%)	Working-line		Near stall	
	100%	55%	100%	55%
$U({\dot{m}}_{\mathrm{cor}})$	$\pm 1.17$	$\pm 7.03$	$\pm 1.97$	$\pm 9.41$
$U(\Pi _{i}^*)$	$\pm 0.84$	$\pm 0.81$	$\pm 0.77$	$\pm 0.81$
$U(\eta _{\mathrm{is,aero},i})$	$\pm 3.42$	$\pm 11.60$	$\pm 3.66$	$\pm 11.73$
$U(\tau )$	$\pm 0.16$	$\pm 0.16$	$\pm 0.16$	$\pm 0.17$

**Fig. 6:**
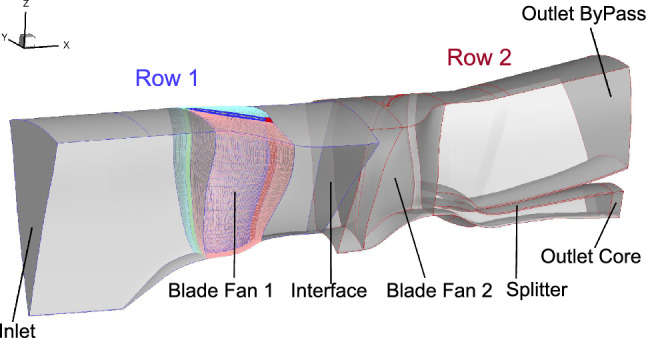
Meshed passage made with AutoGrid with Row 1 in blue contours and Row 2 in red ones

**Fig. 7:**
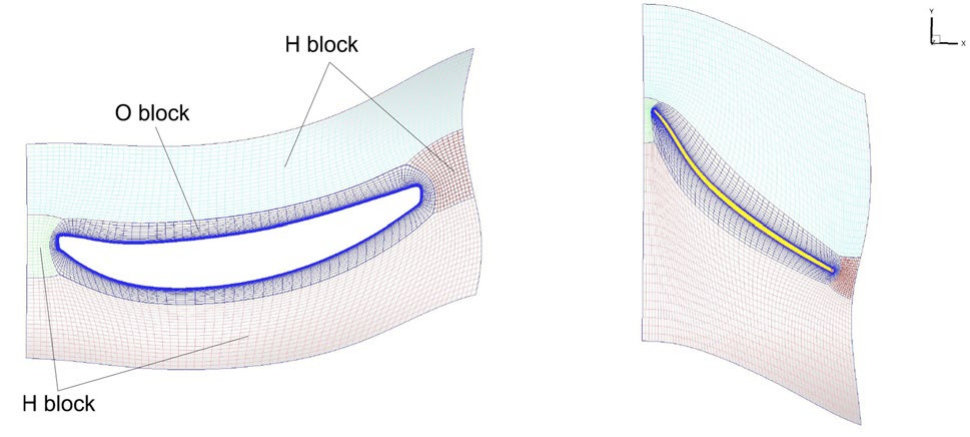
O4H mesh topology at hub (left-hand side) and at tip (right-hand side) of Fan 1 for the medium fidelity grid

*Torque input quantity* The torque measurements for each fan *M*1 and *M*2 were not available for the present study. However, it is known that the torque was measured with T32FNA sensors produced by HBM. Therefore, the measurements come with an $\pm 0.1\%$ interval. Thus, the upper border and the bottom border are calculable, namely, $a_+ = 1.001 M$ and $a_- = 0.999 M$. To have an idea of what would be the uncertainty of the torque ratio $\tau$, the numerical torque values are used instead. To do so, the closest simulations to the experimental data in torque ratio are selected. Then, thanks to the postprocessing, it is possible to determine the torque for each fan. It is important to emphasize here how the input uncertainties are considered. Indeed, when an input quantity appears in the expression of an output quantity, the input uncertainty is calculated by assessing the border values and using Eq. (13). However when the analysis focuses only on the input quantity itself, the input uncertainty is directly provided by the measurement half-width interval and there is no need to use Eq. (13). For instance, the temperature input quantity which is measured with thermocouples of type K within the temperature condition, within the temperature condition $-80^{\circ }\mathrm{C}<T<200^{\circ }$C, its uncertainty is simply $\pm 0.2$ K.

*Uncertainties on the output aerodynamic performances* The iso-torque lines 55%, 90%, 100%, and the iso-speed line 100%* were prioritized for this study, because they represent a large sample of the flight envelope. Thus, they give an idea of the uncertainty of the COBRA fans for a large set of operation. On each of those selected line, only two operating points per iso-line were chosen, namely the Working-line and the Stall ones. Therefore, the expanded uncertainty values of the output measurands: corrected massflow, total pressure ratio, aerodynamic isentropic efficiency, and torque ratio for the 100% and 55% iso-torque lines are given in Table 3 (see Annex for both the 90% iso-torque line and the 100%* iso-speed line in Table 5). Since the infinitesimal massflow data are not known for the present study, it was not possible to integrate both Eqs. (7) and (9) of the respective average total pressure ratio and average isentropic efficiency into the software GUM Workbench. Therefore, regarding the average total pressure ratio $\Pi ^*$, the assumption made is that it has the same uncertainty as the total pressure ratio $\Pi ^*_i$ at the middle of the rake (radial position $R_5$). Those uncertainties could be assimilated, because they are not expected to be different from each other. One possible solution would be to estimate the uncertainty of total pressure ratio at each radial position and to average them, but it would not result in a significant difference. For the average aerodynamic isentropic efficiency $\eta _{\mathrm{is,aero}}$, there is no assumption made. In fact, for the performance maps, it is the mechanical isentropic efficiency $\eta _{\mathrm{is,mech}}$ which is plotted, as stated in Sect. 2.1. The uncertainty of the mechanical isentropic efficiency is directly provided by the CIAM; it is equal to $\pm 0.5\%$. However, the expanded uncertainty on the aerodynamic isentropic efficiency $U(\eta _{\mathrm{is,aero},i})$ is not lost; it is plotted in the radial distributions.

## Numerical modeling

The grids are generated with AutoGrid, the preprocessing is achieved with General Mesh Connector (GMC), and the simulations are run on an High-Performance Computer, named Computer for Advanced Research in Aerospace (CARA), using the in-house developed flow solver TRACE [23].

### Grids

**Fig. 8:**
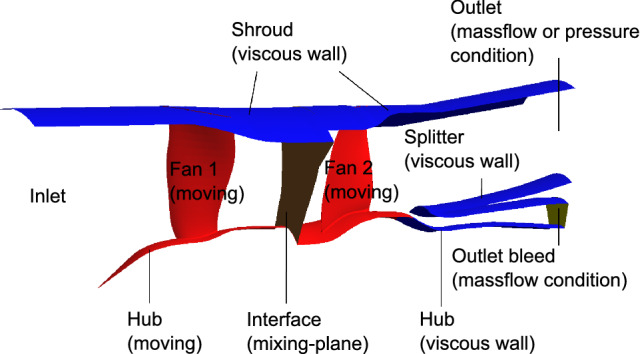
Boundary conditions set with General Mesh Connector on the COBRA architecture

As shown in Fig. 6, the first row includes the inlet and a blade of Fan 1 and the second row includes a blade of Fan 2, the splitter, and the outlets. An O4H topology is used to generate a grid close to the blades (Fig. 7). Figure 7 depicts how the O block and the four H blocks are placed around the blade of Fan 1, with two-dimensional azimuthal views of the mesh topology at the tip and the hub regions. The present study includes a medium and a high fidelity grid, with, respectively, 2.51 and 11.19 million cells. The goal of the medium fidelity grid was to be similar to the grid used during the optimization process and which was generated by G3DHEXA [24]. A mesh sensitivity study has been carried out, during internal previous studies [25], and it showed that the medium fidelity mesh is a time-optimized compromise to perform computational fluid dynamics (CFD) comparisons. However, in the meantime, the high fidelity mesh has been generated to perform future acoustic simulations. Therefore, for validation purposes, it was decided to include that mesh in the present study. The global characteristic values of these two grids are summarized in Table 4.

**Table 4:** Global characteristics of the medium and the high fidelity grids

Fidelity	Medium	High
Cells ($10^6$)	2.51	11.19
Cells row 1 ($10^6$)	1.06	5.47
Cells row 2 ($10^6$)	1.45	5.72
*k*-lines (row 1/row 2) (–)	69/85	141/141
Tip clearance *k*-lines (–)	15	29
Filets *k*-lines (–)	15	25
$y^{+}$ (–)	$>30$ (hub) $<1$ (blades and shroud)	$<1$

### Preprocessing

GMC enables to set the boundary conditions and the system of equations solved by the flow solver afterwards. Figure 8 shows the boundary conditions set for the study. At the inlet, ISA condition of pressure and temperature plus a swirl free inflow are being assumed. The outlet of the core passage is set as a bleed condition, so that the massflow which is leaking out is always specified. The outlet of the bypass passage has either a massflow or a static pressure condition. Preferably, a massflow condition is imposed, so that the experimental bypass massflow values could be used. However, some operating points had struggled to converge, when approaching Stall condition, so a pressure condition is applied instead. Note that the operating points that are simulated with that condition are highlighted with a star. Finally, periodic boundary conditions are imposed along the sides of the passage. The flow solver TRACE computes the steady Reynolds-Averaged Navier–Stokes equations to simulate the supposed ideal gas sucked by the CRTF. To close the Navier–Stokes system of equations, a two-equation model is used [26]. Namely, both the Wilcox 1988 $k-\omega$ and the Shear Stress Transport (SST) turbulence models are used in the present study. For each iso-line, to simulate the experimental operating points, the one with the greatest corrected massflow is the first to be simulated. As soon as that first operating point is converged, the second operating point is simulated by taking the converged simulation, and by adapting the conditions. That process enables to create a numerical iso-line with operating points that are issued from the precedent converged one.

### Postprocessing

**Fig. 9:**
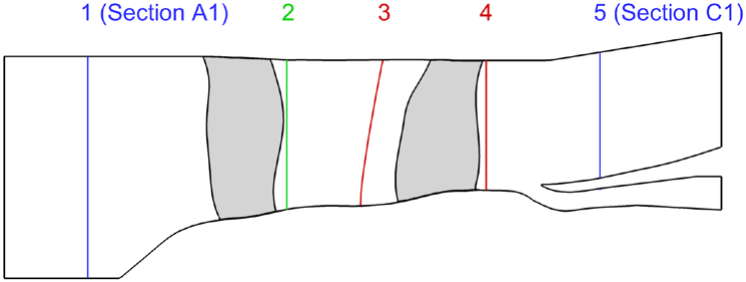
Positions of the postprocessing planes

A simulation is considered as converged if during the last 2000 iterations, the values of corrected massflow, average total pressure ratio, and average aerodynamic isentropic efficiency have stayed in a $\pm 0.1\%$ interval. This criterion is expected to be met for each fan and the complete architecture. Once it is the case, the postprocessing enables to extract and calculate meaningful results from it. Aerodynamic measurements were taken at both A1 and C1 sections. Therefore, the goal was to numerically assess the performance of the CRTF COBRA between the same positions. Therefore, the flow solver calculates the results for the whole domain, but afterwards, it is possible to precise the borders between which the data are extracted and processed. As depicted in Fig. 9, the postprocessing is divided into three domains, namely, the global domain between planes 1 and 5, the Row 1 between planes 1 and 2, and the Row 2 between planes 3 and 4. The postprocessing takes only into account the flow which is going through the bypass passage to assess the fans performances. In addition, the numerical results at each plane are averaged over the height with a massflow method, as performed in the experimental campaign.

## Results

**Fig. 10:**
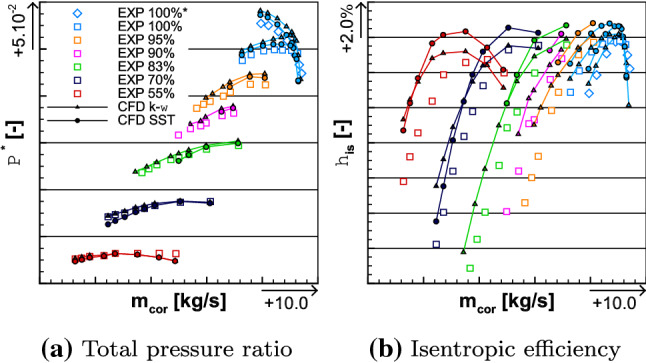
Experimental and numerical performance maps of the CRTF COBRA including the average total pressure ratio (left) and the isentropic efficiency (right)

The experimental campaign enabled to investigate the COBRA fans performances for six different rotating speeds. The performance maps in Fig. 10 depicted of all of them. The 100% running condition is represented in cyan, with the iso-speed line which is symbolized with unfilled-diamond-shape symbols and the iso-torque line with unfilled squares. The 95% (including the Cruise OP), 83% (including the Cutback OP) and 55% (including the Approach OP) running conditions are colorized, respectively, in orange, green, and red. Other running conditions were also tested with the 90% in pink and the 70% in dark blue. The medium fidelity simulations associated with each iso-line are plotted by respecting the color system in place, with the $k-\omega$ turbulence model represented by triangles and the SST one by circles. Among these iso-lines, the compression and efficiency maps from the 100% and 55% are analyzed in Sects. 4.1 and 4.2. They are prioritized, so that one may better understand the aerodynamic performances of the CRTF COBRA at extremal running conditions, that is to say transonic and subsonic flows. Since the observations made for these selected iso-lines can be applied for the rest of the performance map, the detail comparisons of average total pressure ratio, isentropic efficiency, and torque ratio of the other running conditions (70%, 83%, 90%, 95%, and 100%*) are provided in Annex for the sake of clarity.

### Analysis of the 100% iso-torque line

**Fig. 11:**
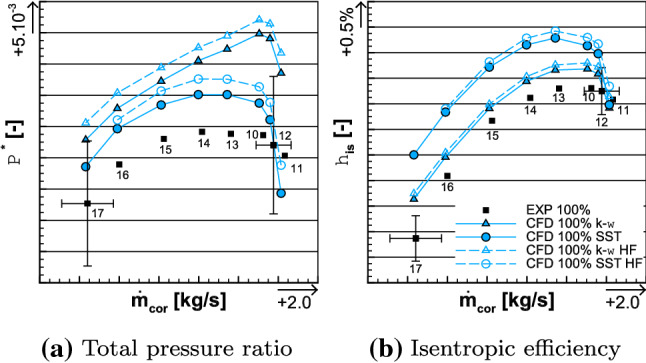
Comparison of the average total pressure ratio (left) and the isentropic efficiency (right) between the experimental and the numerical results for the 100% iso-torque line

**Fig. 12:**
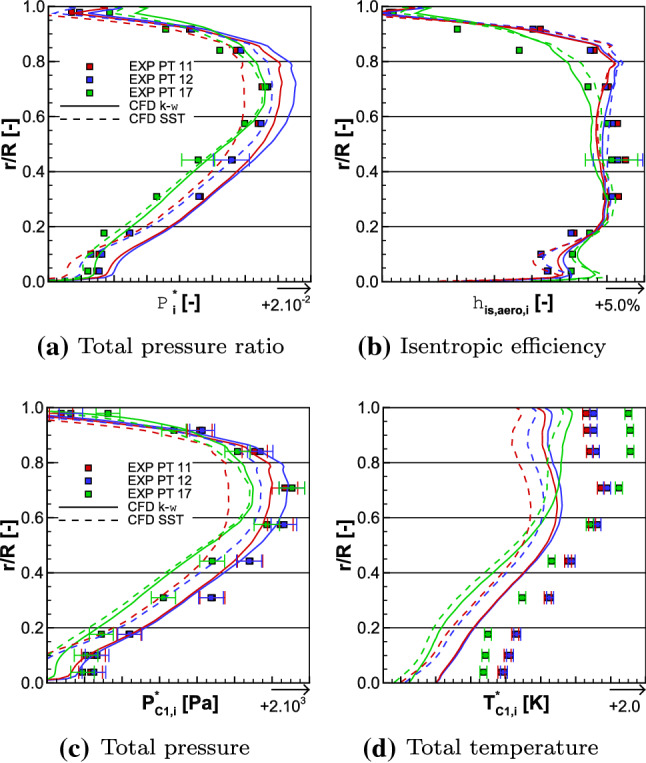
Experimental and numerical radial distributions of total pressure ratio (top left), aerodynamic isentropic efficiency (top right), total pressure (bottom left), and total temperature (bottom right) measured at section C1 for OPs 11, 12, and 17 from the 100% iso-torque line

The iso-torque line 100% is composed of eight operating points as depicted in Fig. 11. The figures represent the average total pressure ratio, the isentropic efficiency, and the torque ratio plotted against the corrected massflow, for both the experimental and numerical results. The black squares represent the experimental values and the cyan lines are the numerical ones. The solid lines with filled symbols are the medium fidelity simulation results and the dashed lines with unfilled symbols are the high fidelity (HF) ones. The distinction between the triangle and circle lines designates, respectively, the $k-\omega$ and SST simulations. The point 12 is close to Aero Design Point conditions, and it is represented with its uncertainties in corrected massflow, total pressure ratio, and isentropic efficiency. They are also plotted for point 17, which is the closest to the Stall operating point. In Fig. 12, the radial distributions of the total pressure ratio, the aerodynamic isentropic efficiency, the total pressure, and the total temperature both measured at section C1 (see plane 5 in Fig. 9) are plotted against the relative height. The experimental operating point data are symbolized with squares namely point 11 (close to Choke operating conditions) in red, point 12 in blue, and point 17 in green, while the numerical results are represented with lines of respective colors. The solid lines represent $k-\omega$ simulations and the dashed ones represent the SST simulations.

#### Observations

The high fidelity radial distributions have not been plotted here; otherwise, the graphs would have been too dense. Indeed, the high fidelity simulations, in Figs. 11 and 17a, show a constant shift, for globally all the operating points, in average total pressure ratio, isentropic efficiency, and torque ratio in comparison to the medium fidelity simulations. At the operating point 12, the shifts are, respectively, about + 0.15%, + 0.21% and + 0.54% for the $k-\omega$ simulations and about + 0.23%, + 0.22% and + 0.58% for the SST ones. For such small differences between the high and medium fidelity simulations and to use the available time efficiently, the medium fidelity set-up is a great compromise (at it was stated in [25] with the mesh sensitivity). Therefore, the present analyses deal with the medium fidelity simulations. The corrected massflow is well estimated over all the operating points with both turbulence models. Indeed, for point 12, the difference in corrected massflow with both models is about $-$ 0.24%, when the uncertainty for this point is $\pm 1.17\%$. For the point 17, the uncertainty is $\pm 1.97\%$ and the difference in corrected massflow is about + 0.14% with both turbulence models.

**Fig. 13:**
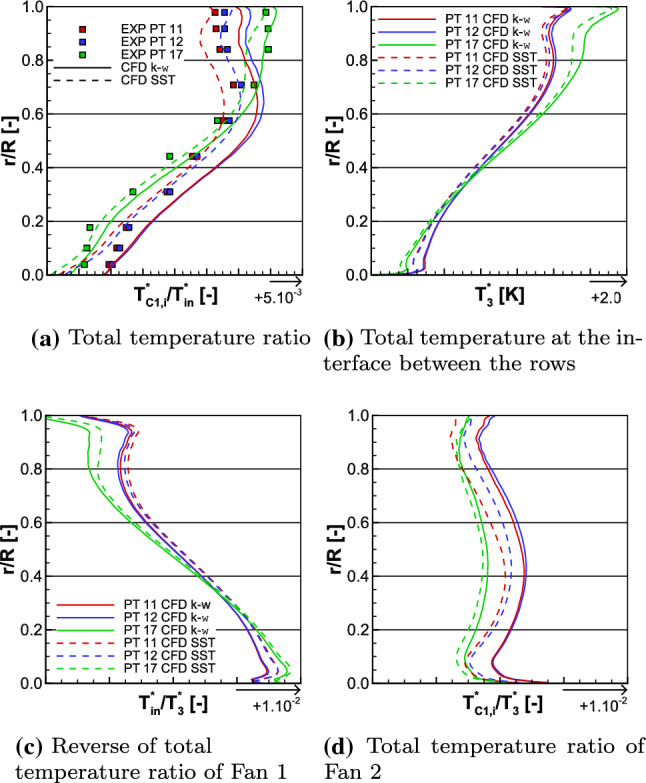
Experimental and numerical radial distributions of total temperature ratio (top left), total temperature at the interface (top right), reverse total temperature ratio of Fan 1 (bottom left), and total temperature ratio of Fan 2 (bottom right) for OPs 11, 12, and 17 from the 100% iso-torque line

Regarding the average total pressure ratio in Fig. 11a, the experimental values are overestimated by the simulations except for the point 11 with the SST turbulence model. For point 12, the SST and $k-\omega$ simulations of the average total pressure ratio are, respectively, 0.31% and 1.31% higher, and for point 17, the SST and the $k-\omega$ simulations are, respectively, 0.47% and 0.78% higher. In Fig. 12a, the radial distributions of total pressure ratio $\Pi ^{*}_i$ are consistent with the average total pressure ratio $\Pi ^{*}$ comparison. Indeed, for each operating point, the numerical radial distributions have higher values than the experimental ones. The only exception is for the simulation SST of operating point 11, but that matches the observation made on the average total pressure ratio. The experimental isentropic efficiency values are better estimated with a $k-\omega$ turbulence model, as can be seen in Fig. 11b. Namely, for operating point 12, the difference between the experimental data and the simulation $k-\omega$ result is about + 0.44% and the uncertainty value is $\pm 0.50\%$, while the SST simulation result is 0.87% higher. For the point 17, none of the difference between the numerical results and the experiment data is within the uncertainty value (also $\pm 0.50\%$); namely + 0.79% for the $k-\omega$ turbulence model and + 1.80% for the SST one. The radial distributions of the aerodynamic isentropic efficiency $\eta _{\mathrm{is,aero},i}$ are consistent with the isentropic efficiency $\eta _{\mathrm{is}}$ observations. Indeed, in Fig. 12b, for all operating points, the aerodynamic isentropic efficiency values of SST simulations are higher than the $k-\omega$ ones. The drop of performance of operating point 17 is due to the losses in the higher part of the blades when approaching Stall conditions. Indeed, numerically as much as experimentally, from relative height 0.6–1.0, the aerodynamic isentropic efficiency is much less for point 17 than any other operating points. In comparison to the point 12, there is about 7.02% less isentropic efficiency between the numerical results at $\frac{r}{R} = 0.8$, and about 11.40% less between the experimental data at the probe $R_{8}$.

#### Interpretation of the observations

As previously highlighted, for all the operating points, the total pressure ratio is higher in the $k-\omega$ simulations than in the SST ones. By analyzing Eq. (5) of the aerodynamic isentropic efficiency and in the hypothetical scenario where the numerical total temperatures $T_{{rm C1},i}^*$ from both the $k-\omega$ and SST simulations would be the same, then it is expected that $\eta _{\mathrm{is,aero},i}$ is also higher in the $k-\omega$ simulations than in the SST ones. Actually that is not the case, it is the reverse; the isentropic efficiency is higher in the SST simulations than in the $k-\omega$ ones. In fact, for the three operating points, it can be seen, in Fig. 12d, that the radial distributions of the total temperature have smaller values in the SST simulations than in the $k-\omega$ ones, also visible in the averaged quantities. This analysis emphasizes the importance of the total temperature in the expression of the isentropic efficiency. The total temperature has also an influence on the torque ratio, which could be expressed as in Eq. (14), with $T_{\mathrm{C1}}^*$ being the massflow average of $T_{\mathrm{C1},i}^*$. It derives from the Euler equation by considering an axial flow that has an average radius

14 $$\begin{aligned} {\tau =-\frac{n_1}{n_2}\cdot \frac{ \frac{T_{\mathrm{C1}}^*}{T_3^*}-1 }{ \frac{T_{\mathrm{in}}^*}{T_3^*}-1 }}. \end{aligned}$$

For each operating point, the rotating velocity ratio $\frac{n_1}{n_2}$ is fixed, and the numerical total temperature at the interface $T_{3}^{*}$ (see plane 3 in Fig. 9) has globally the same value in either a $k-\omega$ or a SST simulation, as can be seen in Fig. 13b. The inlet temperature $T_{\mathrm{in}}^{*}$, being imposed by the inlet boundary condition, is therefore the same no matter the turbulence model, and taken at postprocessing plane 1 (see Fig. 9). Therefore, the radial distributions of the ratio $\frac{T_{\mathrm{in}}^*}{T_3^*}$ are similar, for each operating point, when comparing turbulence models (see Fig. 13c). However, the total temperature $T_{\mathrm{C1},i}^*$ is smaller in the SST simulations than in the $k-\omega$ ones, as mentioned previously. This difference in assessment of total temperature at section C1 is the reason why the torque ratio has different values for the same operating point, when comparing the two turbulence models. Indeed, as can be seen in Fig. 17a, at point 12, the torque ratio of the $k-\omega$ simulation is 5.26% higher than the experimental value; in comparison, it is 1.29% smaller with the SST simulation. At operating point 17, the torque ratio of both simulations is, respectively, 0.71% higher and 1.58% smaller than the experimental data. Therefore, there is a smaller difference in torque ratio values between the $k-\omega$ and the SST simulations at operating point 17 than at 12. For operating point 12, between both turbulence model simulations, there is a constant temperature shift about 2 K, as shown in Fig. 12d. This shift, between the $k-\omega$ and the SST simulations, is propagated to the radial distribution of the total temperature ratio of Fan 2 $\frac{T_{C1}^*}{T_3^*}$, as plotted in Fig. 13d. Consequently, it is also propagated to the the calculation of the torque ratio, with Eq. (14), and that explains the highlighted difference in torque ratio values. In comparison, for operating point 17, there is still a difference in torque ratio but smaller. Indeed, the total temperature $T_{\mathrm{C1},i}^*$ distributions are closer between both turbulence models. Therefore, the difference in total temperature ratio of Fan 2 values between the $k-\omega$ and the SST simulations being smaller, the difference in torque ratio does so.

### Analysis of the 55% iso-torque line

**Fig. 14:**
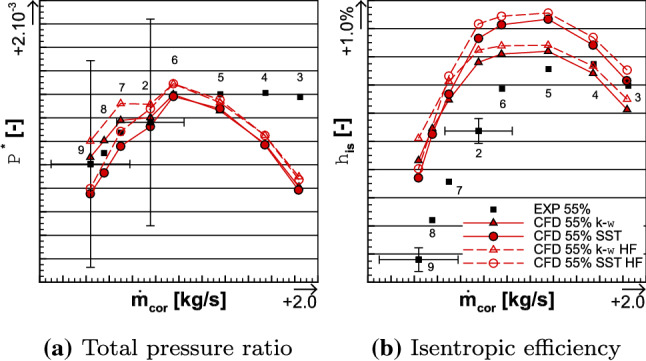
Comparison of the average total pressure ratio (left) and the isentropic efficiency (right) between the experimental and the numerical results for the 55% iso-torque line

In Fig. 13a, the total temperature ratio radial distributions $\frac{T_{\mathrm{C1},i}^*}{T_{in}^*}$ have a constant shift between both turbulence model along the entire relative height, for point 12. It can be deduced that the air in the $k-\omega$ simulation receives more energy by the CRTF, so it has more total enthalpy at the outlet, and hence a higher total temperature. For point 17, the shift is smaller between both turbulence models. However, for that point, it can be stated that the air receives less energy in the higher regions of the blades with the SST simulation, as depicted in Fig. 13a.

**Fig. 15:**
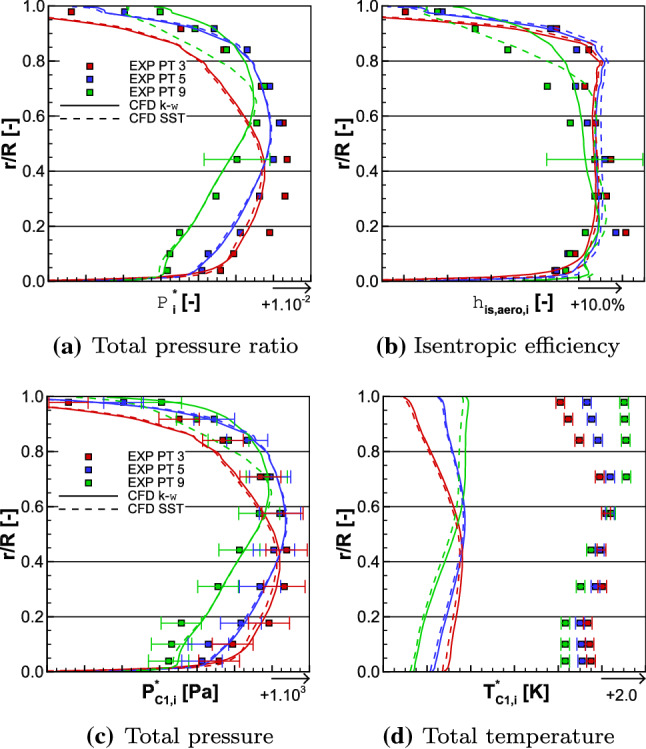
Experimental and numerical radial distributions of total pressure ratio (top left), aerodynamic isentropic efficiency (top right), total pressure (bottom left), and total temperature (bottom right) measured at section C1 for OPs 3, 5, and 9 from the 55% iso-torque line

The iso-torque line 55%, including the Approach running conditions, is also composed of eight operating points and it is depicted in Figs. 14 and 17b. The black squares represent the experimental values and the red lines are the numerical ones. The operating point 2 is on the Working-line, and it is represented with its uncertainties in corrected massflow, total pressure ratio, and isentropic efficiency. These uncertainties are, respectively, plotted for point 9, which is an operating point close to the Stall operating conditions. In Fig. 15, the radial distributions of the total pressure ratio, the aerodynamic isentropic efficiency, the total pressure, and the total temperature both measured at section C1 are plotted against the relative height. The experimental operating point data are symbolized with squares, namely point 3 (close to Choke) in red, point 5 (maximum numerical isentropic efficiency) in blue, and point 9 in green, while the numerical results are represented with lines of respective colors.

#### Observations

In Figs. 14 and 17b, the high fidelity simulations show a constant shift, for globally all the operating points, in total pressure ratio, isentropic efficiency, and torque ratio in comparison to the medium fidelity simulations; respectively, about + 0.09%, + 0.44% and + 0.16% for the $k-\omega$ simulations and about + 0.19%, + 0.55% and + 0.16% for the SST ones. For the same reasons as mentioned in Sect. 4.1, the analyses below only deal with the medium fidelity simulations. The corrected massflows of the eight operating points are well accessed. Indeed, at operating point 2, it is 0.01% smaller for both the $k-\omega$ and SST simulations than the experimental data, while the uncertainty of the corrected massflow at this point is $\pm 7.03\%$. Same conclusion is at operating point 9, where the uncertainty is about $\pm 9.41\%$ and the difference to the experimental value is $-$ 0.06% for both turbulence models.

Regarding the average total pressure ratio, at point 2, the numerical results are within the uncertainty value of $\pm 0.81\%$. Indeed, the $k-\omega$ and SST simulations have, respectively, a difference of + 0.04% and $-$ 0.04% to the experimental data. At point 9, both turbulence model simulations are within the uncertainty value of $\pm 0.81\%$. Indeed, the $k-\omega$ and SST simulations values have, respectively, a difference of + 0.05% and $-$ 0.24% to the experimental data. Over all the operating points, the average total pressure ratio is better estimated at lower corrected massflow. Indeed, at point 3, which has the highest corrected massflow on the iso-torque line, the difference between the experimental data and both the numerical results is $-$ 0.74%. This difference is due to the higher part of the blades. As shown in Fig. 15a, from relative height $\frac{r}{R} = 0.2$ and above, the numerical radial distributions of the total pressure ratio $\Pi ^{*}_{i}$ underestimate the experimental one, at OP 3. For both turbulence models, there are differences about $-$ 0.55% at probe $R_3$ and about $-$ 1.03% at probe $R_8$ in comparison to the experimental data. That observation matches the radial distribution of the total pressure $P_{\mathrm{C1},i}$, as shown in Fig. 15c. Indeed, until relative height $\frac{r}{R}=0.4$ the experimental and numerical total pressures are similar, but above that relative height, they do not match anymore. For both turbulence models, there are differences about $-$ 0.20% at probe $R_3$ and approximately $-$ 0.67% at probe $R_8$ in comparison to the experimental data.

At the maximal isentropic efficiency operating point 5, the average total pressure ratios from both turbulence models differ from the experimental data by only $-$ 0.09%, as plotted in Fig. 14a. This small difference between experimental data and numerical results can also be seen in both total pressure ratio and total pressure radial distributions (Figs. 15a, c). In comparison to the point 3, the radial numerical and experimental values are much closer. For operating point 9 and as highlighted before, the average total pressure ratio from the $k-\omega$ simulation is higher than the experimental data and lower for SST simulation. The reason of this difference also relies on the analysis of the upper part of the blades. Indeed, for both turbulence models, the total pressure ratio radial distributions are similar and they match the experimental data until relative height $\frac{r}{R}= 0.7$. Over this value, the experimental data are underestimated by the SST simulation and overestimated by the $k-\omega$ one.

At both operating points 2 and 9, the uncertainty on the isentropic efficiency is $\pm 0.50\%$. As can be seen in Fig. 14b, the $k-\omega$ and SST simulations values are, respectively, 2.71% and 3.73% higher than the experimental data for point 2, and, respectively, 4.18% and 3.46% higher for point 9. Moreover, for point 3, the isentropic efficiency is higher in the SST simulation than in the $k-\omega$ one. These observations are also noticeable on the radial distribution of the aerodynamic isentropic efficiency $\eta _{\mathrm{is,aero},i}$. Indeed, in Fig. 15b, for operating points 3 and 5, over all the relative heights, the SST simulation values are predominantly higher than the $k-\omega$ ones. In comparison, at OP 9, the difference in isentropic efficiency radial distribution between both turbulence models is more explicit, and it explains why the isentropic efficiency is higher in the simulation $k-\omega$. Until relative height $\frac{r}{R}=0.7$, both numerical results are quiet similar, but above this relative height, the SST simulation issues a greater drop of $\eta _{\mathrm{is,aero},i}$. Once again, this difference is located in the higher part of the blades. As expected, losses and drops of performance in the upper part of the blades characterize the instability limits, when approaching the Stall operating conditions.

The torque ratio is less well assessed in comparison to the iso-torque line 100%, as depicted in Fig. 17b. Indeed, for OP 3, the $k-\omega$ and SST simulation results have a difference of, respectively, $-$ 7.69% and $-$ 9.41% with the experimental data, for OP 5, respectively, $-$ 6.04% and $-$ 6.85%, for OP 2, respectively, $-$ 7.66% and $-$ 8.85%, and for OP 9, respectively, $-$ 8.33% and $-$ 6.36%. However, one may notice that there is less difference between both turbulence models at each operating point, in comparison to the 100% iso-torque line.

**Fig. 16:**
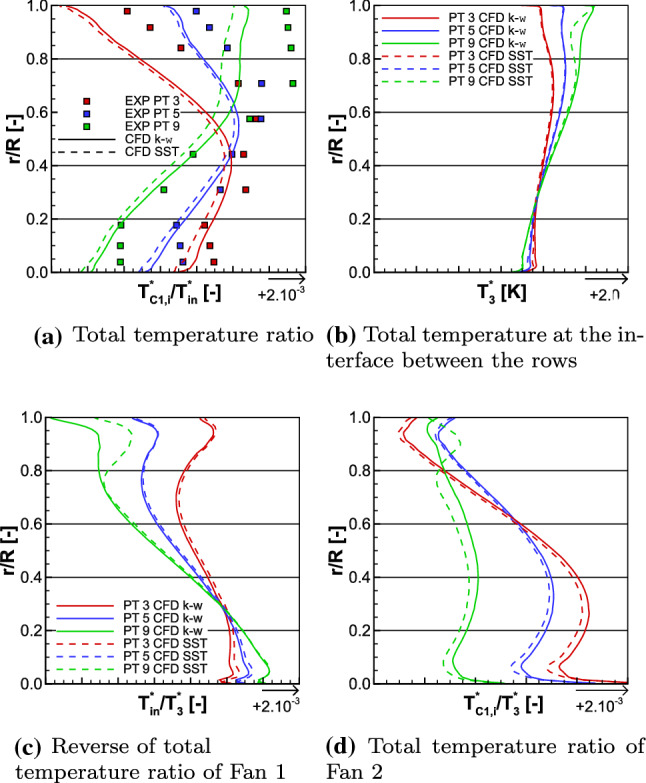
Experimental and numerical radial distributions of total temperature ratio (top left), total temperature at the interface (top right), reverse total temperature ratio of Fan 1 (bottom left), and total temperature ratio of Fan 2 (bottom right) for OPs 3, 5, and 9 from the 55% iso-torque line

#### Interpretation of the observations

It was enlightened in the analysis of the 100% iso-torque line that for the same operating point, between two turbulence model simulations, the bigger the difference in total temperature, the bigger the difference in torque ratio. As shown in the total temperature radial distributions for each operating point, from the iso-torque line 55%, in Figs. 15d and 16b, the respective total temperatures $T_{\mathrm{C1},i}^*$ and $T_{3}^*$ are similar between both turbulence models. For point 9, there is a shift in the upper part of the blades, but it is not constant and does not worth 2 K, like at OP 12 on the 100% iso-torque line. In addition, for each operating point, there is no substantial shift in temperature ratios $\frac{T_{\mathrm{in}}^*}{T_3^*}$ and $\frac{T_{\mathrm{C1}}^*}{T_3^*}$ between turbulence model simulations, as shown, respectively, in Fig. 16c and d. Therefore, at a considered operating point, the estimation of the torque ratio with Eq. (14) does not differ as much as with iso-torque line 100%, from a turbulence model to another. All those elements on the temperature analysis explain why for each operating point, the numerical torque ratio values of the iso-torque line 55% are more similar in comparison to the iso-torque line 100%.

**Fig. 17:**
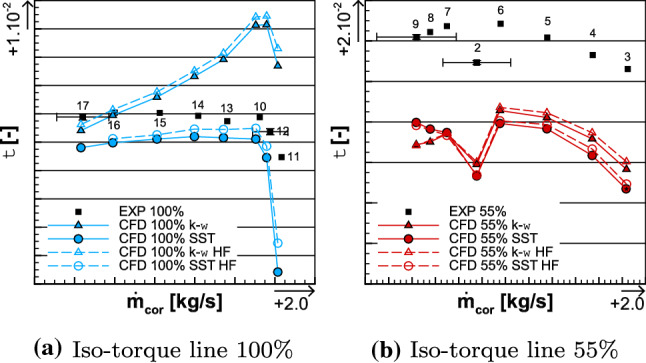
Comparison of the torque ratio between the experimental and the numerical results for both the 100% (left) and 55% (right) iso-torque lines

Furthermore, to explain the reason why there is much more difference between the experimental data and the simulations, in comparison to the iso-torque line 100%, one may compare the total temperature $T_{\mathrm{C1},i}^*$. Indeed, in Fig. 15d along the entire relative height, the simulations have a constant shift about $-$ 6 K, for all three operating points in comparison to the experimental data. That shift is about $-$ 4 K to $-$ 5 K for the iso-torque line 100%, as depicted in Fig. 12d. Therefore, at smaller rotating speed, it results that the transmission of energy to the fluid by the CRTF is less well estimated. That observation could be extended to the total temperature ratio $\frac{T_{\mathrm{C1},i}^*}{T_{in}^*}$ . Indeed, for the iso-torque line 55% in Fig. 16a, that ratio is underestimated by both turbulence models, for all three operating points. In comparison to the iso-torque line 100% with Fig. 13a, where the simulations are much more closer to the experimental data and even higher for the $k-\omega$ ones, for all the three operating points. Therefore, one may assume that since the simulation of the heating is less than what it should, the torque ratio is smaller than what it should, for the iso-torque line 55%. That statement could be enhanced and confirmed with experimental measurements between both fans to evaluate $T^*_3$ , but unfortunately no rakes were placed there during the experimental campaign.

## Conclusion

In the present paper, the studies were carried out only on the iso-torque lines 100% and 55%, but it enabled to draw meaningful remarks and conclusions. The uncertainties study enabled to investigate in more details the measurement methodology and, therefore, enhanced the validation purpose. The flow solver TRACE enables to validate a great majority of the experimental operating points with a massflow boundary condition at the bypass outlet, on the case of the manufactured CRTF COBRA design. A static pressure boundary condition instead helped to explore further the numerical operating limits.

The high fidelity simulations showed negligible differences by considering the difference in time that they required. The drops of performance in total pressure ratio and isentropic efficiency are localized in the higher part of the blades. The study of the sensitivity on the turbulence model shows differences in total pressure ratio and isentropic efficiency which were expected but for the difference in torque ratio. The difference in total temperature ratio, more particularly on the rear fan, between the $k-\omega$ and the SST turbulence models, for the same operating point, is the main reason why there is a difference in torque ratio (Fig. 17).

## Figures and Tables

**Fig. 18:**
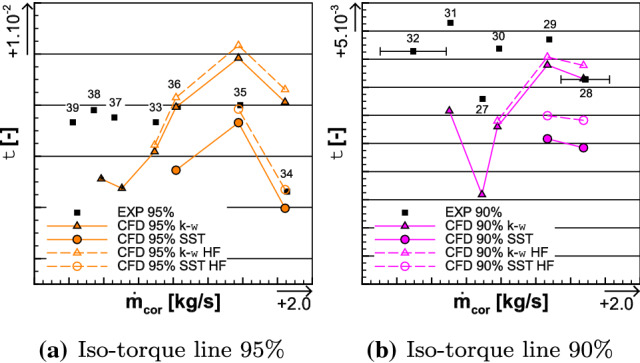
Comparison of the torque ratio between the experimental and the numerical results for both the 95% (left) and 90% (right) iso-torque lines

**Fig. 19:**
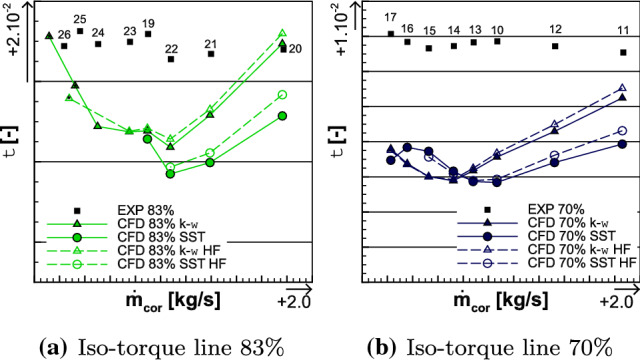
Comparison of the torque ratio between the experimental and the numerical results for both the 83% (left) and 70% (right) iso-torque lines

**Fig. 20:**
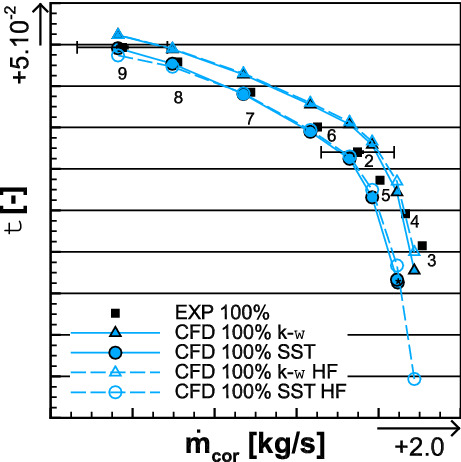
Comparison of the torque ratio between the experimental and the numerical results for the 100%* iso-speed line

**Fig. 21:**
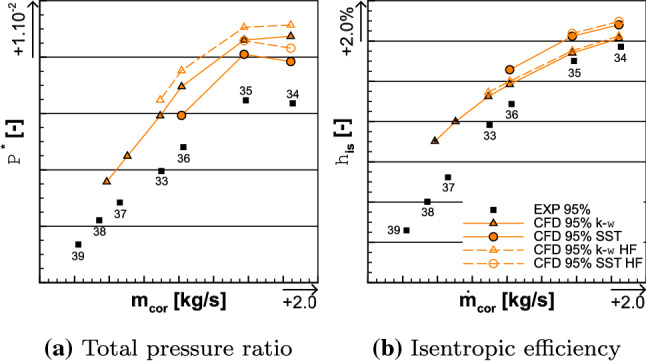
Comparison of the average total pressure ratio (left) and the isentropic efficiency (right) between the experimental and the numerical results for the 95% iso-torque line

**Fig. 22:**
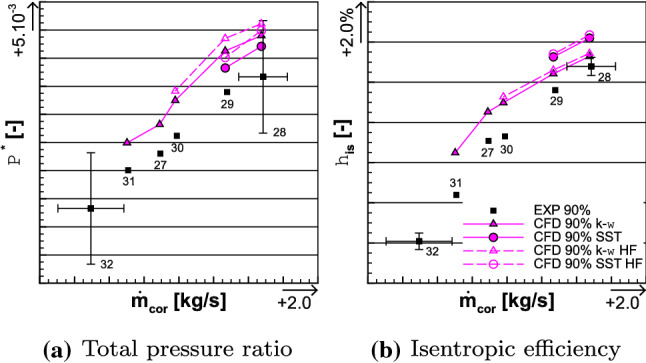
Comparison of the average total pressure ratio (left) and the isentropic efficiency (right) between the experimental and the numerical results for the 90% iso-torque line

**Fig. 23:**
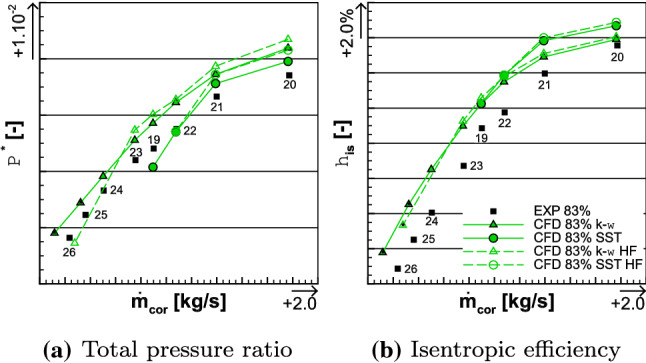
Comparison of the average total pressure ratio (left) and the isentropic efficiency (right) between the experimental and the numerical results for the 83% iso-torque line

**Fig. 24:**
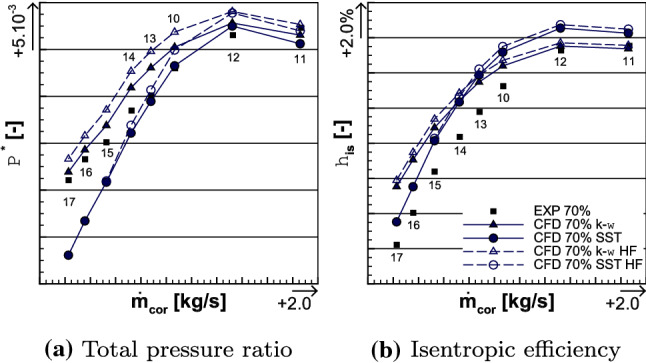
Comparison of the average total pressure ratio (left) and the isentropic efficiency (right) between the experimental and the numerical results for the 70% iso-torque line

**Fig. 25:**
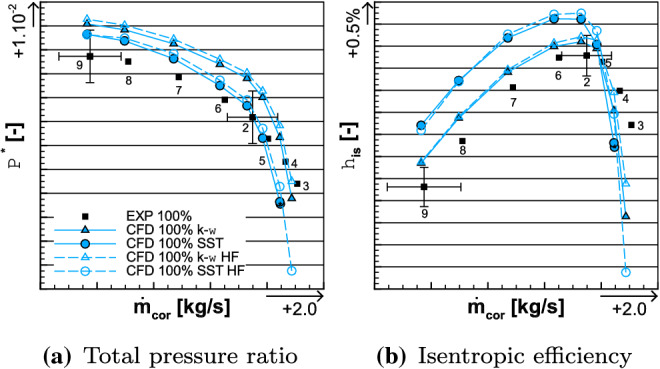
Comparison of the average total pressure ratio (left) and the isentropic efficiency (right) between the experimental and the numerical results for the 100%* iso-speed line

## Annex

Functional relationships used for the uncertainty estimations

15 $$\begin{aligned} {\dot{m}}_{\mathrm{cor}}&=f(P_0, T_0, P_{\mathrm{s,A1}}, {{\dot{m}}}_{\mathrm{Core}}, P_{\mathrm{ISA}}, T_{\mathrm{ISA}}, S_{\mathrm{A1}}, \gamma , r, K) \end{aligned}$$

16 $$\begin{aligned} \Pi _i^*&=f(P_{\mathrm{C1},i}^*, P_0, T_0, P_{\mathrm{s,A1}}, {{\dot{m}}}_{\mathrm{Core}}, S_{\mathrm{A1}}, \gamma , r, K) \end{aligned}$$

17 $$\begin{aligned} \eta _{\mathrm{is,aero},i}&=f(P_{\mathrm{C1},i}^*, T_{\mathrm{C1},i}^*, P_0, T_0, P_{\mathrm{s,A1}}, {{\dot{m}}}_{\mathrm{Core}}, S_{\mathrm{A1}},\gamma , r, K) \end{aligned}$$

18 $$\begin{aligned} \tau&=f(M_1, M_2). \end{aligned}$$

**Table 5:** Corrected massflow, total pressure ratio, aerodynamic isentropic efficiency, and torque ratio

Speed (%)	Working-line		Near stall	
	100%*	90%	100%*	90%
$U({\dot{m}}_{\mathrm{cor}})$	$\pm 1.18$	$\pm 2.17$	$\pm 1.59$	$\pm 3.48$
$U(\Pi _{i}^*)$	$\pm 0.84$	$\pm 0.81$	$\pm 0.83$	$\pm 0.81$
$U(\eta _{\mathrm{is,aero},i})$	$\pm 3.43$	$\pm 4.28$	$\pm 3.25$	$\pm 4.62$
$U(\tau )$	$\pm 0.16$	–	$\pm 0.16$	–

See Table 5 and Figs. 18, 19, 20, 21, 22, 23, 24, 25.

## Abbreviations

ACARE: Advisory Council for Aviation Research
CARA: Computer for Advanced Research in Aerospace
COBRA: Counter rotating fan system for high bypass ratio aircraft engine
COVID: COronaVIrus Disease
CRTF: Counter rotating turbo fan
DLR: German Aerospace Center
GMC: General mesh connector
ISA: International Standard Atmosphere
OP: Operating point
RPM: Revolutions per minute
TCS: Turbulence control screen
UHBR: Ultra high bypass ratio

## List of symbols and indices

$$S\, ({\mathrm{m}}^{2})$$: Area
$$K\,$$ (–): Discharge coefficient
$$f\,$$ (–): Functional relationship
$$x\,$$ (–): Input quantity
$$M_a\,$$ (–): Mach number
$${\dot{m}}\, (\mathrm{kg/s})$$: Massflow
$$y\,$$ (–): Output measurand
$$r\, (\mathrm{m})$$: Radial position
$$R_i\, (\mathrm{m})$$: Probe position
$$i\,$$ (–): Reduction coefficient
$n\, RPM$: Revolutions per minute
$c_p\, (\mathrm{J/(kg K)})$: Specific heat capacity
$$M\, (\mathrm{N}\, \mathrm{m})$$: Torque
$$P\, (\mathrm{Pa})$$: Total pressure
$$T\, (\mathrm{K})$$: Total temperature
$$\eta \,$$ (–): Efficiency
$\gamma \,$ (–): Heat capacity ratio
$$\varDelta \,$$ (–): Power losses
$$\omega \, (\mathrm{rad/s})$$: Rotation speed
$$\tau \,$$ (–): Torque ratio
$\Pi \,$ (–): Total pressure ratio
$$()_{\mathrm{in}}$$: Inlet
$$()_{0}$$: Ambient
$$()_{\mathrm{is}}$$: Isentropic
$$()_{\mathrm{mech}}$$: Mechanic
